# Benchmarking large language models for pathogen–disease classification in post-acute infection syndromes

**DOI:** 10.1093/bib/bbag089

**Published:** 2026-03-06

**Authors:** Syed Mohammed Khalid, Tom Wölker, Leidy-Alejandra G Molano, Simon Graf, Andreas Keller

**Affiliations:** Clinical Bioinformatics, Saarland University, 66123 Saarbrücken, Germany; Clinical Bioinformatics, Saarland University, 66123 Saarbrücken, Germany; Clinical Bioinformatics, Saarland University, 66123 Saarbrücken, Germany; Helmholtz Institute for Pharmaceutical Research Saarland (HIPS)–Helmholtz Centre for Infection Research (HZI), Saarland University Campus, 66123 Saarbrücken, Germany; Clinical Bioinformatics, Saarland University, 66123 Saarbrücken, Germany; Helmholtz Institute for Pharmaceutical Research Saarland (HIPS)–Helmholtz Centre for Infection Research (HZI), Saarland University Campus, 66123 Saarbrücken, Germany; Clinical Bioinformatics, Saarland University, 66123 Saarbrücken, Germany; Helmholtz Institute for Pharmaceutical Research Saarland (HIPS)–Helmholtz Centre for Infection Research (HZI), Saarland University Campus, 66123 Saarbrücken, Germany

**Keywords:** post-acute infection syndromes (PAIS), pathogen–disease associations, large language models (LLMs), zero-shot learning, few-shot learning, chain-of-thought prompting, reasoning models, text classification

## Abstract

Post-Acute Infection Syndromes (PAIS) are medical conditions that persist following acute infections from pathogens such as SARS-CoV-2, Epstein–Barr virus, and Influenza virus. Despite growing global awareness of PAIS and the exponential increase in biomedical literature, only a small fraction of this literature pertains specifically to PAIS, making the identification of pathogen–disease associations within such a vast, heterogeneous, and unstructured corpus a significant challenge for researchers. This study evaluated the effectiveness of large language models (LLMs) in extracting these associations through a binary classification task using a curated dataset of 1000 manually labeled PubMed abstracts. We benchmarked a wide range of open-source LLMs of varying sizes (4B–70B parameters), including generalist, reasoning, and biomedical-specific models. We also investigated the extent to which prompting strategies such as zero-shot, few-shot, and Chain of Thought (CoT) methods can improve classification performance. Our results indicate that model performance varied by size, architecture, and prompting strategy. Zero-shot prompting produced the most reliable results: Mistral-Small-Instruct-2409 and Llama-3.1-Nemotron-70B-Instruct achieved balanced accuracy scores of 0.81 and 0.80, respectively, along with macro-F1 scores of up to 0.80, while maintaining minimal invalid outputs. While few-shot and CoT prompting often degraded performance in generalist models, reasoning models such as DeepSeek-R1-Distill-Llama-70B and QwQ-32B demonstrated improved accuracy and consistency when provided with additional context.

## Introduction

Traditionally, acute infectious diseases were perceived as short-lived episodes that ended either in patient recovery or death [1]. However, now they are growingly being recognized for their potential to lead to chronic or prolonged symptoms known as post-acute infection syndromes (PAIS), which refer to the diverse set of persistent conditions following acute infection [1]. Following the COVID-19 pandemic, approximately 65 million individuals experienced ongoing symptoms lasting at least three months post-infection [2, 3]. Chronic sequelae have been documented following infections with a wide spectrum of pathogens, including SARS-CoV-2, Epstein–Barr virus, Influenza virus, and Ebola virus [4–7]. The nonspecific nature of PAIS symptoms, along with the absence of reliable biomarkers, poses substantial challenges for clinical diagnosis, treatment, and the development of preventive strategies. Although biomedical research output has grown substantially in recent years, only a limited portion of studies have systematically addressed PAIS [8]. As a result, researchers face considerable challenges in identifying clear pathogen–disease associations from the existing largely unstructured literature base. To accelerate discovery in this field, there is a pressing need for scalable, systematic approaches that can synthesize existing knowledge and uncover novel insights beyond the limitations of manual and labor-intensive methods.

Given these challenges, early efforts to automate the extraction of pathogen–disease associations relied on sentence-level co-occurrence. An ontology-based text-mining study extracted 3420 pathogen–disease pairs from 1.8 million open-access articles, reporting 64% precision, 89% recall, and an F-score of 0.74 [9]. However, the system accepted only associations occurring at least ten times and with a Normalized Point-wise Mutual Information (NPMI) $\geq 0.2$, thereby discarding many rare but clinically relevant links. Additionally, sentence-level co-occurrence further introduced false positives, often due to abbreviation ambiguities [9]. Such rigid thresholds are especially problematic in contexts where relevant pathogen–disease links are often reported using heterogeneous and evolving terminology.

Generative artificial intelligence (AI) systems have raised considerable interest due to their rapidly expanding capabilities and significant potential across a diverse range of applications [10]. In biomedicine, recent methods have used supervised machine learning to study biomedical associations by combining transformer-based sequence encoders with graph attention mechanisms and by constructing consensus representations over multiple biological interaction graphs [11, 12]. Within language-based AI systems, large language models (LLMs), particularly foundation models built on transformer architectures [13] and optimized using reinforcement learning [14], such as GPT-4o from OpenAI [15], Llama 3 from Meta [16], or reasoning models like DeepSeek-R1 [17], have emerged as particularly influential, demonstrating impressive performance in diverse tasks such as comprehension [18], summarization [19], text-classification [20], and information retrieval [21].

Despite their observed general capabilities, questions remain regarding the effectiveness of LLMs within the biomedical research domain. Past research presents a conflicting set of approaches and outcomes. On one hand, domain-specific pretrained language models such as BioBERT [22], when fine-tuned on biomedical datasets, have consistently outperformed generalist LLMs, including GPT-4, its predecessors, and Flan-T5 in tasks such as biomedical text classification and reasoning [23, 24]. On the other hand, some studies suggest that generalist models, when coupled with innovative prompting techniques, can rival or even surpass the performance of specialized models [25, 26]. Importantly, the apparent differences reported across prior biomedical LLM evaluations are closely tied to the characteristics of the underlying tasks and corpora, particularly in settings such as PAIS.

Prompting, in the context of language models, refers to the input crafted to steer the model’s generated output. A model’s performance on a given task can be significantly affected by the way the prompt is formulated, sometimes in unexpected or surprising ways [27]. A wide range of prompting approaches has been explored, indicating significant potential in improving LLMs’ performance in biomedical-related tasks [28]. For example, advanced prompting methods such as few-shot learning and retrieval-augmented reasoning have been proven effective in biomedical literature analysis, particularly for entity recognition tasks [23, 29, 30]. In contrast, in structured biomedical classification tasks, simple prompting strategies have been observed to outperform more complex reasoning techniques such as chain-of-thought (CoT) or retrieval-augmented generation (RAG) [31]. Similar observations were made in other studies, where incorporating external evidence into prompts was found to negatively impact model accuracy [32].

Despite extensive research in biomedical language processing, most existing studies focus on general biomedical tasks or conventional disease contexts. Notably, to our existing knowledge, there is a lack of benchmarking efforts evaluating LLMs for identifying pathogen–disease relationships, particularly within the unique literature of PAIS. Most existing benchmarks focus on settings in which relevant biomedical relationships are explicitly stated and supported by clear evidence. In contrast, PAIS research is marked by non-specific and varied clinical manifestations, heterogeneity in study designs and data collection, and an incomplete understanding of underlying mechanisms, which hinder consistent understanding and comparison between studies [8].To address this gap, we systematically benchmarked state-of-the-art LLMs to assess their reliability in accurately identifying pathogen–disease associations in PAIS-related biomedical literature. We employed various prompting strategies, including Zero-shot, Few-shot, and Chain-of-Thought (CoT) methods, to evaluate and compare models ranging from small language models (SLMs) to those with 70 billion parameters. Additionally, we analyzed performance differences between generalist foundation, reasoning, and domain-specific models.

## Methods

### Dataset

Given the absence of publicly available labeled datasets specifically focused on pathogen–disease relationships within the context of post-acute infection syndromes (PAIS), we constructed an evaluation dataset of PubMed titles and abstracts tailored to this task. We began by first compiling lists of pathogens, diseases, and their relationships from PathoPhenoDB [33] (accessed February 15, 2024), Disbiome [34] (accessed February 19, 2024), gcPathogen [35] (accessed January 15, 2024), and DO [36] (August 2024 release). The pathogens and diseases extracted from the sources were normalized with NCBI Taxonomy Database [37] for the pathogens and DO for the diseases and pathogen and disease synonyms were extracted as well. From the cross product of the pathogens and diseases we created PubMed queries, excluding any pathogen–disease combinations where a relationship was extractable (applicable sources were PathoPhenoDB, DisBiome) or considered “similar”. We define similarity as either the pathogen or the disease term being contained within the other (e.g. “porcine diarrhea virus” and “diarrhea”), or having an edit distance of 0.85 or greater (e.g. “Leishmania” and “leishmaniasis,” where the larger term was truncated by three characters to set a stricter threshold). The PubMed queries consisted of the pathogen and disease terms from the sources, as well as available synonyms with the PubMed Filter [TIAB] for each term, fha [FILT] to include only citations with abstracts and the [Date - Publication] between 2004 and 2024/02/29. The queries were split up into batches of 10 000 queries and retrieved using (Entrez Direct [38] v.16.2). If the original query failed the query was repeated without the pathogen and disease synonyms. Next, the query results were refined per batch, using the NCBI Taxonomy to identify results which are part of the same pathogen lineage for each disease. The result with the most detailed lineage was kept and the others were discarded as duplicates. For example, if an abstract for the same disease returned results with both the pathogens “Rickettsia” (genus) and “Rickettsia africae” (species) only “Rickettsia africae” (the more specific term) was kept. From this refined set of results, we selected 1000 entries randomly and uniformly. Finally, the labels for each entry in the dataset were assigned manually according to the following specified set of rules (An abstract was labeled as indicating a relationship if it fulfilled any one of Criteria 1–4):

1. Does the abstract investigate the pathogen and report evidence for the disease?
2. Does the abstract investigate the disease and report evidence for the pathogen?
3. Does the abstract focus on the relation between the Pathogen and the Disease?
4. Does the abstract state the relation between Pathogen and Disease, but does not focus on it?

The final dataset comprised 1000 PubMed title–abstract pairs. We used 994 of the samples for evaluation and reserved 6 samples for few-shot prompting. The dataset consists of 70% negative and 30% positive examples. The dataset further included abstracts spanning three relation categories—spurious, not significant, and significant—reflecting varying degrees of reported association strength. To assess annotation reliability, a second annotator re-labeled a randomly selected subset of 200 abstracts using the same labeling criteria. Inter-annotator agreement was quantified using both percent agreement and Cohen’s $\kappa$. The annotators agreed on 75% of cases, corresponding to a Cohen’s $\kappa$ of 0.50, indicating moderate agreement.

- Spurious: Abstracts with a spurious relation between the disease and the pathogen term.
- Not Significant: Abstracts where the authors investigate the relation between disease and pathogen, but the pathogen did not show any significant results.
- Significant: Abstracts where the authors investigate the relation between disease and pathogen, and have significant results.

Of the 1000 abstracts, 130 (13.0%) contained sufficient evidence for annotators to assign relationship qualifiers. These were most commonly annotated as significant (67 abstracts; 6.7% of the dataset), followed by spurious (51; 5.1%) and not significant (12; 1.2%). All remaining abstracts were not assigned additional qualifiers, as they did not contain explicit evidence supporting any of these categories.

### Models

We evaluated a diverse set of open-weight LLMs hosted on Hugging Face, restricted to those feasible to run on 2 $\times$ NVIDIA H100 80 GB GPUs. Our selection covers multiple scales and architectures (Table 1). For clarity and readability, we retain the full model names (including the “-Instruct” suffix when applicable) in all evaluation tables, plots, and this section, while using shortened names in the main text. When a shortened name is used, it refers to the specific variant listed in the Table 1.

**Table 1:** LLM Specifications.

**Model**	**Parameters**	**Domain**
Llama-3.1-70B-Instruct	70B	General
Llama-3.3-70B-Instruct	70B	General
Llama-3.1-Nemotron-70B-Instruct	70B	General
Qwen2.5-72B-Instruct	72B	General
Mistral-Small-Instruct-2409	22B	General
Phi-3.5-MoE-Instruct	42B	General
Llama-3.1-8B-Instruct	8B	General
Llama-3-8B-Instruct	8B	General
Qwen2.5-7B-Instruct	7B	General
Ministral-8B-Instruct-2410	8B	General
Phi-4-Mini-Instruct	4B	General
QwQ-32B	32B	Reasoning
DeepSeek-R1-Distill-Llama-70B	70B	Reasoning
DeepSeek-R1-Distill-Llama-8B	8B	Reasoning
DeepSeek-R1-Distill-Qwen-7B	7B	Reasoning
DeepSeek-R1-Distill-Qwen-32B	32B	Reasoning
BioMistral-7B	7B	Bio
PMC-LLaMA	13B	Bio
Meditron-70B	70B	Bio

#### Generalist foundation models

We considered models across three size ranges. **Large (70B+):** Llama-3.3-70B-Instruct, Llama-3.1-70B-Instruct, NVIDIA’s Llama-3.1-Nemotron-70B-Instruct (an RLHF-tuned Llama variant), and Qwen2.5-72B-Instruct, all strong generalist performers [16, 39, 40]. **Mid-sized (24–42B):** Phi-3.5-MoE-Instruct and Mistral-Small-Instruct-2409 [41, 42]. **Small ($\leq 8B$):** Llama-3.1-8B-Instruct, Llama-3-8B, Qwen2.5-7B-Instruct, Ministral-8B-Instruct-2410, and Phi-4-Mini-Instruct [16, 40, 42, 43]. These models were included to assess latency–accuracy trade-offs.

#### Reasoning models

We evaluated distilled variants of DeepSeek R1 (DeepSeek-R1-Distill-Llama-70B/8B and DeepSeek-R1-Distill-Qwen-32B/7B), proposed to retain the reasoning capabilities of the DeepSeek-R1 model [17], along with QwQ-32B, another reasoning model [44].

#### Biomedical models

Given the biomedical focus of our research, we include three domain-specific models: BioMistral-7B, Meditron-70B (trained on curated medical corpora), and PMC-LLaMA-13B (trained on 4.8M biomedical papers and instruction-tuned) [45–47]. These models were selected based on their strong performance on the PubMedQA benchmark and to ensure diversity in model size and training scope [48].

### Prompting strategies

#### Zero-Shot

Zero-shot prompting requires no domain-specific training data; instead, the model is given a task description and generates predictions based on prior knowledge [49, 50]. Effective prompts typically include (1) task instruction, (2) input text (abstract and title), and (3) constraints on output format [51].

**Figure 1:**
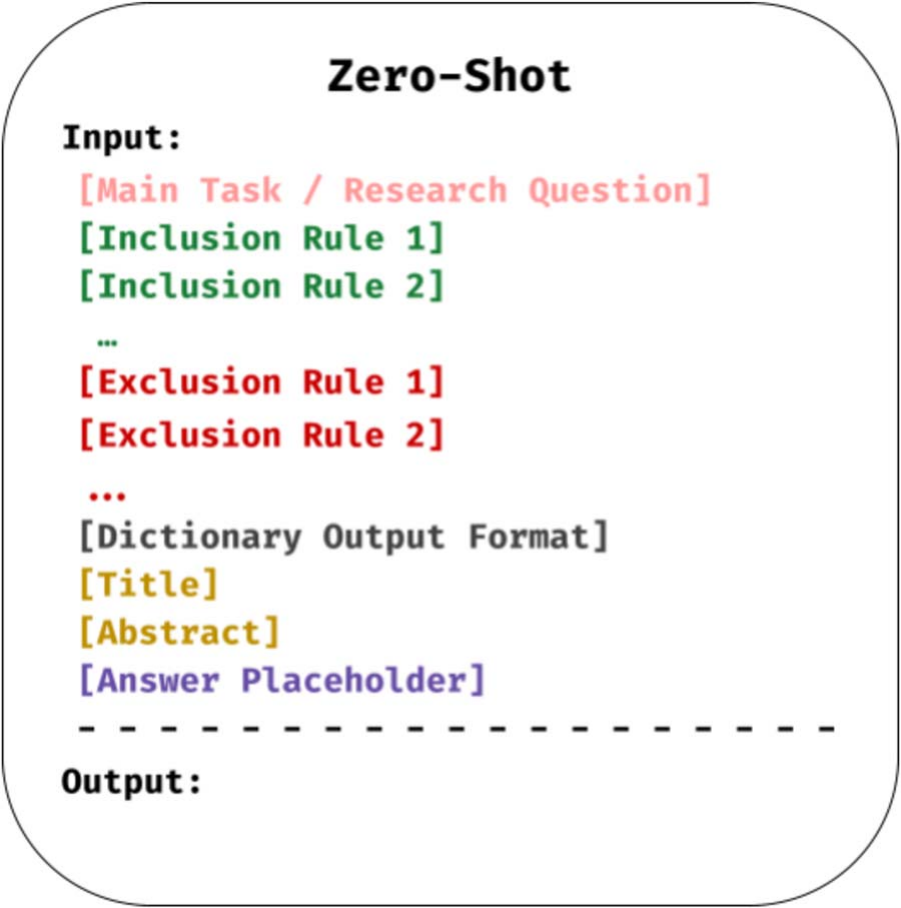
Zero-shot prompt structure.

Considering our classification task involves assisting the model in reviewing a particular abstract and its title, we note that this is a common practice in abstract screening for systematic review and meta-analysis studies. We review previous studies on LLM-based approaches for abstract screening and replicate the prompting strategy they propose, closely following one established method while adhering to rules outlined in prior work [28, 51]. Hence, while constructing the prompts, we adopted a standardized approach to mimic a typical interaction between a senior researcher and a research assistant. To ensure consistency, we required outputs in a fixed dictionary format {“unrelated”: 0, “relationship”: 1} or {“unrelated”: 1, “relationship”: 0}. The decision to restrict the model to only output a dictionary format was to combat the challenge posed by the LLMs free-text nature, hence in the case where the model tends to be more verbose, it largely includes a dictionary containing the final result in the response, we can then proceed to extract the verdict using custom parsing scripts. Figure 1 shows the prompt structure used.

#### Few-Shot

In few-shot prompting, models are provided with a small number of input–output examples to illustrate the task, in contrast to zero-shot prompting where no examples are given [51]. In our study, few-shot prompting was used to assess whether limited in-context supervision could improve pathogen–disease classification performance.

**Figure 2:**
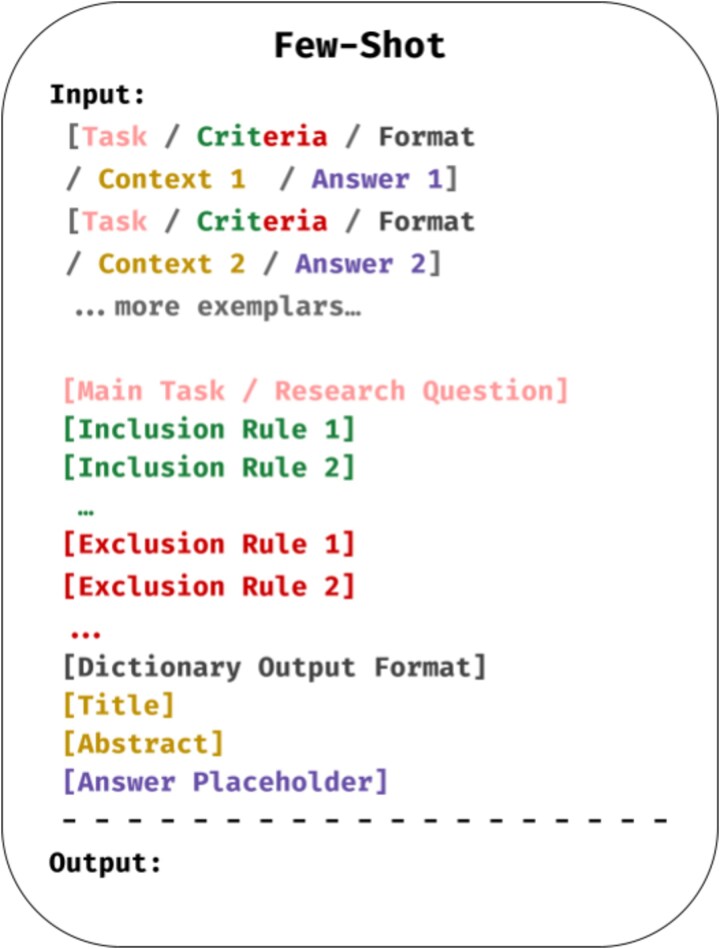
Few-shot prompt structure.

We adopted both 3-shot and 6-shot settings and selected a small number of representative abstracts from each of the three dataset-defined relation categories (spurious, not significant, and significant), ensuring balanced coverage across categories. The six examples used for prompting were excluded from evaluation. Figure 2 illustrates the prompt structure used.

#### Chain-of-Thought

**Figure 3:**
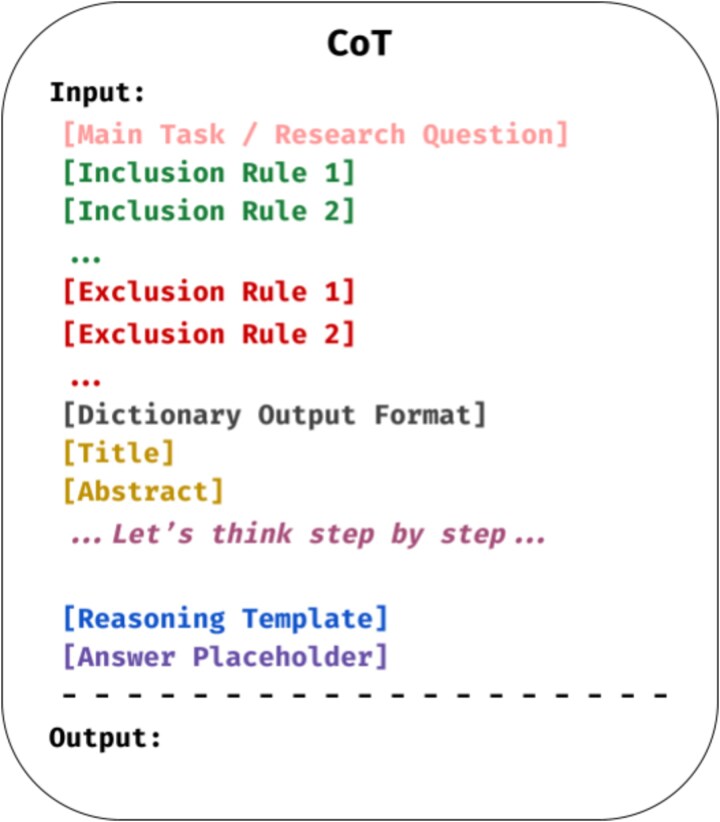
Chain-of-Thought (CoT) prompt structure.

Chain-of-Thought (CoT) was introduced as a technique to prompt LLMs in a way that allows a step-by-step reasoning process, and this is seen to have generated more accurate results. Originally demonstrated for a given math problem, the prompt would show the reasoning process step by step, forcing the model to mimic how humans break down problems into logical intermediate steps [50]. The following is the prompt we used to enable a step-by-step reasoning process before the model makes any predictions. We intentionally omitted a few-shot exemplars in the CoT prompt to isolate the effect of verbalized reasoning. Figure 3 illustrates the prompt structure used.

### Evaluation

Evaluating LLM outputs is difficult due to their free-text nature, which often produces noisy or partially correct responses. To address this, we constrained models to a fixed dictionary format and parsed outputs with custom scripts. Any response that did not match the expected dictionary pattern was flagged as invalid and logged.

We evaluated the results under two views: (i) strict, counting invalid outputs as incorrect, and (ii) lenient, excluding them. Performance was measured using Avg. Query Latency, Balanced Accuracy, Precision, Recall, Macro-F1, Specificity, MCC, and Invalid Output rate. Confidence intervals were estimated via 2000 bootstrap resamples.

All generations used greedy decoding (temperature=0, seed=42). The default maximum output cap was set to 300 tokens, increased to 1000 for Qwen-72B, CoT, and reasoning models to avoid truncation. Prompting strategies varied by model: generalist and reasoning models were tested with zero-, few-shot (3/6-shot), and CoT prompting, while biomedical models were restricted to zero-shot due to their smaller context windows.

## Results

We evaluate LLMs on a classification task: determining whether a given title and abstract state a pathogen–disease relationship. Predictions were scored under two policies: (i) lenient, where invalid outputs are excluded, and (ii) strict, where they are counted as negatives. The lenient view forms the basis of our analysis as it isolates classification ability, while strict results are reported for selected models to test robustness to format constraints.

### Supervised and heuristic baseline models

Before evaluating large language models, we established non-LLM baseline performance using rule-based heuristics, classical machine-learning methods, and a transformer-based pretrained language model.

Specifically, we evaluated: (i) rule-based approaches based on entity existence and token-level proximity between pathogen and disease mentions, (ii) a TF-IDF + logistic regression classifier trained using stratified k-fold cross-validation, and (iii) a fine-tuned BioBERT model trained under standard supervised settings using stratified k-fold cross-validation.

These models were evaluated using the same metrics as the LLMs, allowing us to contextualize zero-shot LLM performance relative to traditional supervised pipelines. The performance of all heuristic and supervised baseline models is summarized in Table 2.

**Table 2:** Performance comparison of heuristic, supervised baseline, and best-performing zero-shot approaches.

**Model**	**Precision**	**Recall**	**Macro F1**	**Specificity**	**Balanced Accuracy**	**MCC**
Existence-Based	0.36	0.88	0.41	0.19	0.54	0.10
Proximity-Based	0.42	0.34	0.55	0.75	0.55	0.10
TF-IDF + LogReg	0.51	0.51	0.63	0.75	0.63	0.26
BioBERT	0.52	0.55	0.64	0.73	0.64	0.28
**Mistral-Small-Instruct-2409**	0.69	0.82	**0.80**	0.81	**0.81**	**0.61**

Bold values highlight the best-performing approach for the selected key metrics (Macro F1, Balanced Accuracy, and MCC).

### Baseline Zero-shot performance

Having established supervised and heuristic baselines, we evaluated 19 open-weight LLMs under zero-shot prompting (Table 3). Mistral-Small and Qwen2.5-72B formed the top tier, both achieving balanced accuracy of approximately 0.81. While Qwen2.5-72B achieved a high balanced accuracy, it produced invalid outputs (1.1%), whereas Mistral-Small was fully robust (0% invalids; MCC=0.61).

**Table 3:** Performance of LLMs under zero-shot prompting.

**Model**	**Precision**	**Recall**	**Macro F1**	**Specificity**	**Balanced Accuracy**	**MCC**	**Invalid Outputs (%)**	**Avg. Query Time (s)**
**Qwen2.5-72B-Instruct**	0.70 (0.67-0.76)	0.79 (0.76-0.85)	**0.80 (0.78-0.83)**	0.83 (0.80-0.86)	**0.81 (0.79-0.84)**	**0.60 (0.56-0.67)**	1.11%	3.71
Llama-3.1-70B-Instruct	0.53 (0.51-0.56)	0.96 (0.95-0.99)	0.70 (0.67-0.73)	0.56 (0.52-0.60)	0.76 (0.74-0.79)	0.51 (0.48-0.56)	0.00%	0.39
Llama-3.3-70B-Instruct	0.53 (0.51-0.56)	0.95 (0.93-0.98)	0.69 (0.67-0.72)	0.56 (0.52-0.60)	0.75 (0.73-0.78)	0.50 (0.46-0.54)	0.00%	0.52
**Llama-3.1-Nemotron-70B-Instruct**	0.60 (0.57-0.63)	0.91 (0.89-0.95)	**0.75 (0.73-0.78)**	0.68 (0.63-0.71)	**0.80 (0.77-0.82)**	**0.56 (0.52-0.61)**	0.00%	0.39
DeepSeek-R1-Distill-Llama-70B	0.57 (0.54-0.60)	0.90 (0.88-0.95)	0.73 (0.70-0.76)	0.64 (0.60-0.68)	0.77 (0.75-0.80)	0.52 (0.48-0.58)	0.00%	0.29
QwQ-32B	0.52 (0.51-0.57)	0.79 (0.76-0.86)	0.68 (0.66-0.72)	0.63 (0.59-0.67)	0.71 (0.69-0.75)	0.39 (0.36-0.48)	1.91%	0.30
DeepSeek-R1-Distill-Qwen-32B	0.53 (0.51-0.56)	0.95 (0.93-0.98)	0.69 (0.67-0.72)	0.56 (0.51-0.60)	0.75 (0.73-0.78)	0.50 (0.46-0.55)	0.00%	0.05
Phi-3.5-MoE-Instruct	0.51 (0.49-0.54)	0.91 (0.87-0.94)	0.67 (0.64-0.70)	0.54 (0.50-0.59)	0.73 (0.70-0.75)	0.44 (0.39-0.49)	0.70%	3.24
**Mistral-Small-Instruct-2409**	0.69 (0.65-0.72)	0.82 (0.77-0.86)	**0.80 (0.77-0.82)**	0.81 (0.77-0.84)	**0.81 (0.78-0.84)**	**0.61 (0.54-0.65)**	0.00%	0.20
DeepSeek-R1-Distill-Qwen-7B	0.34 (0.35-0.35)	1.00 (0.99-1.00)	0.26 (0.26-0.27)	0.00 (0.00-0.01)	0.50 (0.50-0.50)	0.01 (-0.05-0.06)	0.00%	0.05
DeepSeek-R1-Distill-Llama-8B	0.40 (0.39-0.41)	0.97 (0.94-0.98)	0.47 (0.44-0.49)	0.23 (0.20-0.26)	0.60 (0.58-0.62)	0.26 (0.21-0.29)	0.00%	0.79
BioMistral-7B	0.36 (0.36-0.37)	0.97 (0.94-0.98)	0.35 (0.33-0.38)	0.10 (0.08-0.13)	0.53 (0.52-0.55)	0.12 (0.07-0.17)	0.00%	0.07
Llama-3-8B-Instruct	0.65 (0.61-0.70)	0.58 (0.52-0.62)	0.71 (0.68-0.74)	0.84 (0.81-0.87)	0.71 (0.67-0.73)	0.43 (0.36-0.49)	0.00%	0.33
Phi-4-Mini-Instruct	0.48 (0.45-0.51)	0.75 (0.69-0.78)	0.63 (0.60-0.65)	0.58 (0.53-0.61)	0.66 (0.63-0.68)	0.31 (0.24-0.35)	2.72%	0.32
Qwen2.5-7B-Instruct	0.51 (0.49-0.56)	0.63 (0.58-0.69)	0.65 (0.62-0.68)	0.69 (0.66-0.73)	0.66 (0.63-0.69)	0.31 (0.25-0.37)	3.22%	0.19
Ministral-8B-Instruct-2410	0.43 (0.42-0.45)	0.96 (0.93-0.98)	0.55 (0.52-0.58)	0.34 (0.31-0.38)	0.65 (0.63-0.67)	0.34 (0.29-0.37)	0.00%	0.05
Llama-3.1-8B-Instruct	0.66 (0.62-0.71)	0.66 (0.60-0.70)	0.74 (0.71-0.77)	0.82 (0.79-0.85)	0.74 (0.71-0.77)	0.48 (0.42-0.54)	0.00%	0.15
Meditron-70B	0.40 (0.39-0.42)	0.90 (0.87-0.94)	0.49 (0.47-0.53)	0.29 (0.25-0.33)	0.60 (0.57-0.63)	0.22 (0.17-0.28)	2.52%	3.72
PMC-LLaMA	0.35 (0.21-0.51)	0.05 (0.03-0.08)	0.43 (0.41-0.46)	0.95 (0.93-0.97)	0.50 (0.49-0.52)	0.00 (-0.06-0.08)	17.10%	0.96

Bold values highlight the best-performing model(s) for the selected key metrics (Macro F1, Balanced Accuracy, and MCC).

**Figure 4:**
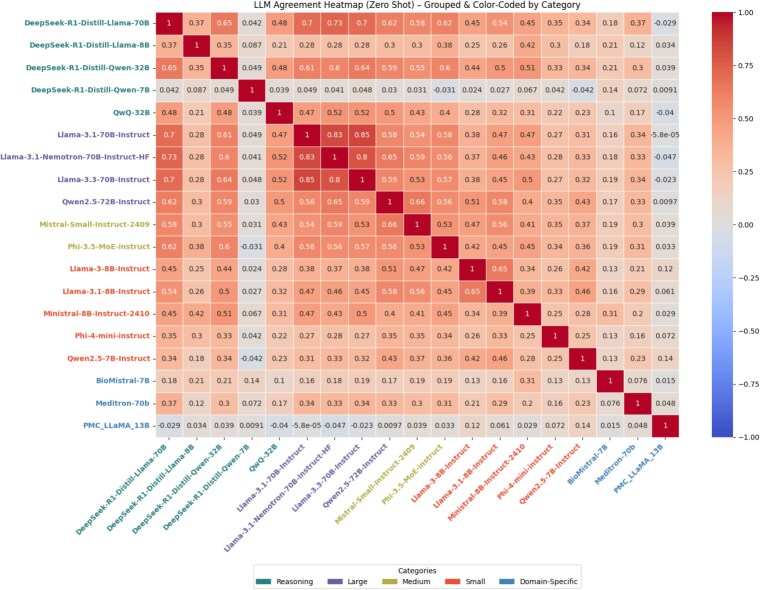
Zero-shot LLM agreement heatmap grouped by model category.

Llama-3.1-Nemotron-70B showed comparable performance (balanced accuracy = 0.80, MCC = 0.56), and other Llama-family models exhibited similar precision–recall balance, consistent with the inter-model agreement patterns shown in Fig. 4. Paired bootstrap analysis (2,000$\times$) 95% confidence intervals (CIs) on $\Delta$-Balanced-Accuracy confirmed that Qwen2.5-72B trails both Llama-3.1-Nemotron-70B (($\Delta$ = –0.041, 95% CI = [–0.064, –0.018]) and Mistral ($\Delta$ = –0.059, 95% CI = [–0.087, –0.031]), whereas Mistral-Small and Llama-3.1-Nemotron-70B form a statistical tie ($\Delta$ = +0.018, CI –0.010 $\ldots$ +0.046).

### Effect of prompting strategies on performance

Building on the zero-shot baseline, we evaluated the impact of few-shot (3- and 6-shot) and Chain-of-Thought (CoT) prompting on model performance.

#### Few-Shot

**Figure 6:**
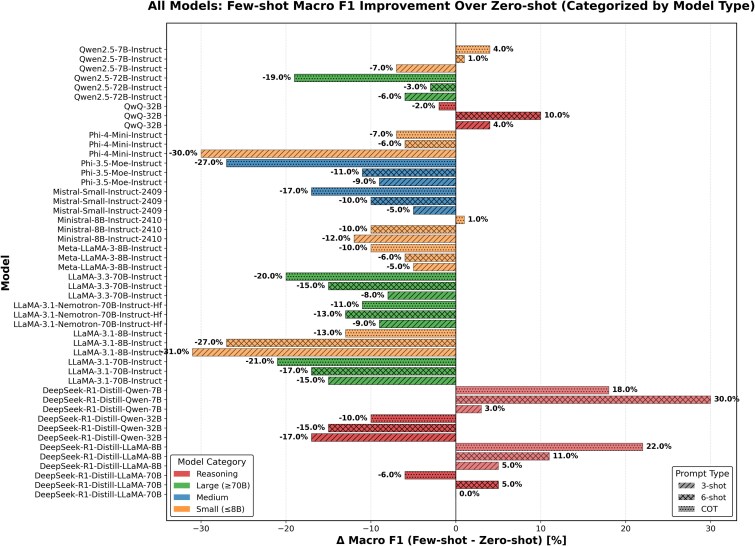
Macro-F1 change relative to zero-shot performance across models.

Few-shot prompting degraded performance for most generalist models (Fig. 6). High-performing zero-shot models such as Qwen2.5-72B and Mistral-Small showed reduced accuracy and increased invalid outputs when examples were added (Tables 4, 5). For instance, Qwen2.5-72B exhibited substantial performance degradation under 3-shot prompting, accompanied by a sharp rise in invalid outputs (10.8%). Similar declines were observed across Llama-family models and Phi-3.5-MoE, indicating that in-context examples often harmed robustness and precision.

**Table 4:** Performance of LLMs under 3-shot prompting.

**Model**	**Precision**	**Recall**	**Macro F1**	**Specificity**	**Balanced Accuracy**	**MCC**	**Invalid Outputs (%)**	**Avg. Query Time (s)**
**Qwen2.5-72B-Instruct**	0.63 (0.60-0.67)	0.75 (0.69-0.79)	**0.74 (0.71-0.77)**	0.76 (0.72-0.79)	**0.75 (0.72-0.78)**	**0.49 (0.43-0.55)**	10.76%	8.04
Llama-3.1-70B-Instruct	0.44 (0.43-0.46)	0.98 (0.96-0.99)	0.55 (0.52-0.58)	0.34 (0.29-0.37)	0.66 (0.63-0.67)	0.36 (0.31-0.39)	0.00%	1.14
Llama-3.3-70B-Instruct	0.47 (0.46-0.50)	0.92 (0.88-0.94)	0.61 (0.59-0.65)	0.45 (0.41-0.49)	0.69 (0.65-0.71)	0.38 (0.32-0.42)	0.70%	7.19
Llama-3.1-Nemotron-70B-Instruct	0.51 (0.49-0.54)	0.94 (0.91-0.96)	0.66 (0.63-0.69)	0.52 (0.47-0.55)	0.73 (0.70-0.75)	0.46 (0.40-0.50)	0.00%	0.89
**DeepSeek-R1-Distill-Llama-70B**	0.57 (0.55-0.61)	0.87 (0.83-0.91)	**0.73 (0.70-0.76)**	0.66 (0.61-0.69)	**0.77 (0.73-0.79)**	**0.51 (0.45-0.56)**	0.00%	12.59
QwQ-32B	0.56 (0.55-0.60)	0.93 (0.89-0.95)	0.72 (0.70-0.75)	0.62 (0.58-0.66)	0.77 (0.75-0.80)	0.53 (0.48-0.57)	0.80%	9.06
DeepSeek-R1-Distill-Qwen-32B	0.42 (0.42-0.45)	0.98 (0.96-0.99)	0.52 (0.49-0.55)	0.30 (0.25-0.33)	0.64 (0.62-0.66)	0.33 (0.29-0.36)	0.00%	0.61
Phi-3.5-MoE-Instruct	0.44 (0.43-0.47)	0.85 (0.80-0.88)	0.58 (0.55-0.61)	0.43 (0.39-0.47)	0.64 (0.61-0.66)	0.28 (0.22-0.33)	3.62%	7.63
**Mistral-Small-Instruct-2409**	0.63 (0.59-0.67)	0.78 (0.73-0.83)	**0.75 (0.72-0.78)**	0.75 (0.71-0.78)	**0.77 (0.73-0.79)**	**0.52 (0.45-0.57)**	0.00%	0.56
DeepSeek-R1-Distill-Qwen-7B	0.35 (0.35-0.36)	0.99 (0.98-1.00)	0.29 (0.28-0.31)	0.04 (0.02-0.05)	0.51 (0.51-0.52)	0.09 (0.04-0.13)	0.00%	0.13
DeepSeek-R1-Distill-Llama-8B	0.39 (0.39-0.43)	0.75 (0.71-0.81)	0.52 (0.50-0.56)	0.40 (0.38-0.46)	0.57 (0.56-0.62)	0.15 (0.11-0.24)	0.30%	3.05
Llama-3-8B-Instruct	0.51 (0.47-0.53)	0.85 (0.80-0.88)	0.66 (0.62-0.68)	0.57 (0.52-0.60)	0.71 (0.67-0.73)	0.40 (0.33-0.44)	0.20%	0.89
Phi-4-Mini-Instruct	0.35 (0.35-0.36)	0.95 (0.93-0.97)	0.33 (0.31-0.35)	0.08 (0.06-0.11)	0.52 (0.50-0.53)	0.06 (0.00-0.12)	3.22%	0.93
Qwen2.5-7B-Instruct	0.52 (0.45-0.57)	0.34 (0.27-0.37)	0.58 (0.55-0.61)	0.83 (0.81-0.87)	0.58 (0.55-0.61)	0.19 (0.12-0.25)	2.82%	0.52
Ministral-8B-Instruct-2410	0.39 (0.38-0.40)	0.99 (0.97-1.00)	0.43 (0.40-0.45)	0.18 (0.15-0.20)	0.58 (0.56-0.60)	0.24 (0.20-0.27)	0.00%	0.16
Llama-3.1-8B-Instruct	0.38 (0.37-0.39)	0.94 (0.91-0.96)	0.43 (0.40-0.46)	0.20 (0.17-0.23)	0.57 (0.54-0.59)	0.18 (0.12-0.23)	3.52%	0.93

Bold values highlight the best-performing model(s) for the selected key metrics (Macro F1, Balanced Accuracy, and MCC).

**Table 5:** Performance of LLMs under 6-shot prompting.

**Model**	**Precision**	**Recall**	**Macro F1**	**Specificity**	**Balanced Accuracy**	**MCC**	**Invalid Outputs (%)**	**Avg. Query Time (s)**
**Qwen2.5-72B-Instruct**	0.67 (0.64-0.72)	0.77 (0.72-0.82)	**0.77 (0.75-0.81)**	0.80 (0.77-0.84)	**0.79 (0.76-0.82)**	**0.55 (0.51-0.62)**	5.53%	13.77
Llama-3.1-70B-Instruct	0.42 (0.42-0.45)	0.98 (0.96-0.99)	0.53 (0.51-0.56)	0.31 (0.27-0.35)	0.64 (0.62-0.66)	0.33 (0.30-0.37)	0.00%	1.91
Llama-3.3-70B-Instruct	0.42 (0.41-0.45)	0.90 (0.86-0.93)	0.54 (0.51-0.57)	0.35 (0.31-0.39)	0.62 (0.60-0.65)	0.26 (0.21-0.32)	2.52%	13.15
Llama-3.1-Nemotron-70B-Instruct	0.47 (0.47-0.51)	0.94 (0.92-0.97)	0.62 (0.60-0.66)	0.45 (0.42-0.50)	0.70 (0.68-0.73)	0.40 (0.37-0.46)	0.00%	1.91
**DeepSeek-R1-Distill-Llama-70B**	0.64 (0.62-0.68)	0.87 (0.83-0.91)	**0.78 (0.76-0.81)**	0.74 (0.71-0.78)	**0.80 (0.78-0.83)**	**0.58 (0.54-0.64)**	0.00%	19.50
**QwQ-32B**	0.64 (0.61-0.68)	0.88 (0.86-0.92)	**0.78 (0.76-0.81)**	0.74 (0.70-0.77)	**0.81 (0.79-0.84)**	**0.59 (0.55-0.64)**	1.01%	14.88
DeepSeek-R1-Distill-Qwen-32B	0.43 (0.43-0.46)	0.97 (0.95-0.99)	0.54 (0.52-0.58)	0.33 (0.29-0.37)	0.65 (0.63-0.67)	0.34 (0.31-0.38)	0.00%	1.03
Phi-3.5-MoE-Instruct	0.44 (0.43-0.46)	0.94 (0.92-0.97)	0.56 (0.53-0.59)	0.37 (0.32-0.40)	0.66 (0.63-0.68)	0.34 (0.29-0.38)	0.20%	11.72
Mistral-Small-Instruct-2409	0.55 (0.53-0.59)	0.75 (0.71-0.80)	0.70 (0.67-0.73)	0.68 (0.64-0.72)	0.72 (0.69-0.75)	0.41 (0.36-0.47)	0.20%	1.12
DeepSeek-R1-Distill-Qwen-7B	0.42 (0.40-0.45)	0.75 (0.71-0.80)	0.56 (0.54-0.60)	0.47 (0.43-0.51)	0.61 (0.58-0.65)	0.21 (0.16-0.28)	0.00%	0.08
DeepSeek-R1-Distill-Llama-8B	0.45 (0.43-0.46)	0.94 (0.91-0.96)	0.58 (0.55-0.61)	0.40 (0.36-0.44)	0.67 (0.65-0.69)	0.35 (0.31-0.40)	0.20%	1.04
Llama-3-8B-Instruct	0.50 (0.47-0.52)	0.76 (0.71-0.80)	0.65 (0.62-0.68)	0.60 (0.57-0.64)	0.68 (0.65-0.71)	0.34 (0.28-0.40)	0.50%	1.45
Phi-4-Mini-Instruct	0.43 (0.41-0.46)	0.80 (0.77-0.85)	0.57 (0.55-0.61)	0.45 (0.42-0.50)	0.63 (0.61-0.66)	0.25 (0.21-0.32)	0.00%	0.47
Qwen2.5-7B-Instruct	0.53 (0.49-0.56)	0.64 (0.59-0.69)	0.66 (0.63-0.69)	0.70 (0.66-0.74)	0.67 (0.64-0.70)	0.33 (0.27-0.39)	1.91%	0.79
Ministral-8B-Instruct-2410	0.39 (0.38-0.40)	0.99 (0.97-1.00)	0.45 (0.42-0.48)	0.21 (0.18-0.25)	0.60 (0.58-0.62)	0.26 (0.23-0.30)	0.00%	0.26
Llama-3.1-8B-Instruct	0.38 (0.37-0.40)	0.89 (0.85-0.92)	0.47 (0.45-0.51)	0.27 (0.25-0.31)	0.58 (0.56-0.61)	0.18 (0.13-0.24)	3.82%	1.45

Bold values highlight the best-performing model(s) for the selected key metrics (Macro F1, Balanced Accuracy, and MCC).

In contrast, reasoning models consistently benefited from few-shot prompting. DeepSeek-Llama-70B and QwQ-32B showed steady improvements in balanced accuracy, macro-F1, and MCC with increasing prompt depth, while maintaining low invalid output rates. DeepSeek-Qwen-32B represented a partial exception, achieving very high recall at the expense of specificity, leading to less reliable predictions. A small subset of models exhibited inconsistent performance trends with prompt depth; for example, Phi-4-Mini declined under 3-shot prompting but partially recovered under 6-shot, without exceeding its zero-shot baseline.

Overall, few-shot prompting failed to deliver consistent gains across models and frequently increased invalid outputs, whereas reasoning models formed a clear exception, demonstrating improved performance with added context.

#### Chain-of-Thought

Overall, CoT prompting degraded performance for most generalist models, mainly by reducing specificity (shifting predictions toward positives) and increasing invalid outputs. For example, Qwen2.5-72B dropped from balanced accuracy 0.81 (MCC 0.60) to 0.68 (MCC 0.34) with invalids rising to 25.8%, and Llama-3.1-70B showed a strong specificity collapse (0.56$\rightarrow$0.26), lowering balanced accuracy (0.76$\rightarrow$0.62) and MCC (0.51$\rightarrow$0.31).

**Figure 5:**
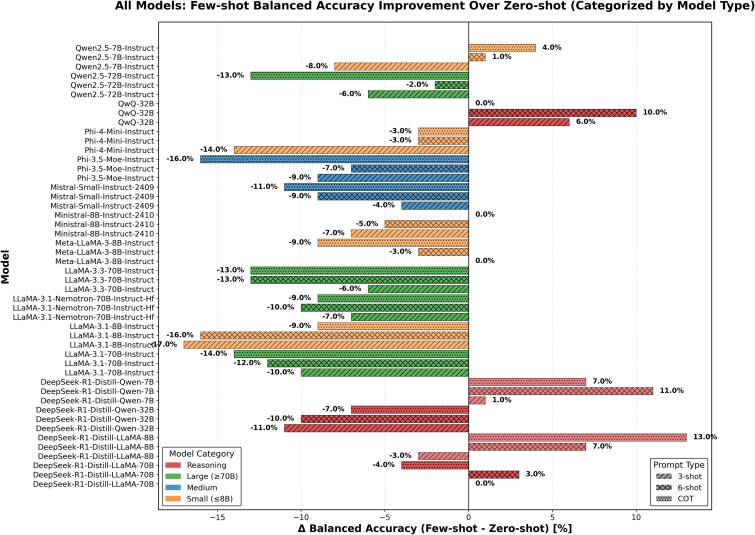
Balanced accuracy change relative to zero-shot performance across models.

After additionally applying CoT to reasoning models, the pattern became mixed but more robust: QwQ-32B stayed essentially unchanged (balanced accuracy 0.71; MCC 0.39$\rightarrow$0.41), while smaller DeepSeek-R1 distills benefited (e.g. DeepSeek-R1-Distill-Llama-8B: balanced accuracy 0.60$\rightarrow$0.73; MCC 0.26$\rightarrow$0.43) (Table 6), largely by improving specificity. Larger reasoning distills did not consistently improve. Thus, CoT is generally detrimental for generalist models, but comparatively stable for reasoning models (Fig. 5).

**Table 6:** Performance of LLMs under CoT prompting.

**Model**	**Precision**	**Recall**	**Macro F1**	**Specificity**	**Balanced Accuracy**	**MCC**	**Invalid Outputs (%)**	**Avg. Query Time (s)**
Qwen2.5-72B-Instruct	0.46 (0.42- 0.50)	0.86 (0.82-0.90)	0.61 (0.59- 0.65)	0.49 (0.45-0.53)	0.68 (0.64 -0.70)	0.34 (0.28-0.40)	25.80%	12.24
Llama-3.1-70B-Instruct	0.41 (0.40-0.42)	0.99 (0.97-1.00)	0.49 (0.45-0.52)	0.26 (0.21-0.28)	0.62 (0.60-0.64)	0.31 (0.26-0.33)	0.10%	3.49
Llama-3.3-70B-Instruct	0.41 (0.40-0.42)	0.98 (0.96-0.99)	0.49 (0.46-0.52)	0.26 (0.21-0.28)	0.62 (0.59-0.63)	0.29 (0.25-0.33)	0.00%	5.62
**Llama-3.1-Nemotron-70B-Instruct**	0.49 (0.47-0.51)	0.94 (0.92-0.97)	0.64 (0.61-0.67)	0.48 (0.43-0.52)	**0.71 (0.68-0.74)**	0.42 (0.38-0.47)	0.00%	5.47
**DeepSeek-R1-Distill-Llama-70B**	0.51 (0.47-0.55)	0.92 (0.89-0.94)	0.67 (0.64-0.70)	0.54(0.50-0.57)	**0.73 (0.70-0.75)**	0.45 (0.40-0.49)	0.00%	12.39
**QwQ-32B**	0.51 (0.47-0.55)	0.87 (0.83-0.90)	0.66 (0.63-0.69)	0.56 (0.52-0.60)	**0.71 (0.70-0.75)**	0.41 (0.40-0.49)	0.20%	3.61
DeepSeek-R1-Distill-Qwen-32B	0.45 (0.42-0.49)	0.96 (0.94-0.98)	0.59 (0.56-0.62)	0.39 (0.35-0.42)	0.68 (0.66-0.70)	0.38 (0.33-0.42)	0.00%	3.50
Phi-3.5-MoE-Instruct	0.38 (0.37-0.39)	0.98 (0.96-1.00)	0.40 (0.37-0.43)	0.15 (0.11-0.17)	0.57 (0.55-0.58)	0.20 (0.15-0.24)	0.00%	11.95
Mistral-Small-Instruct-2409	0.48 (0.46-0.51)	0.92 (0.90-0.96)	0.63 (0.60-0.66)	0.48 (0.42-0.51)	0.70 (0.67-0.73)	0.39 (0.36-0.45)	0.00%	0.20
DeepSeek-R1-Distill-Qwen-7B	0.38 (0.35-0.42)	0.93 (0.91-0.96)	0.44 (0.41-0.47)	0.21 (0.18-0.24)	0.57 (055-0.59)	0.19 (0.14-0.23)	0.00%	0.66
DeepSeek-R1-Distill-Llama-8B	0.54 (0.49-0.58)	0.82 (0.89-0.94)	0.69 (0.66-0.71)	0.63 (0.60-0.67)	0.73 (0.70-0.75)	0.43 (0.37-0.48)	0.00%	1.18
Llama-3-8B-Instruct	0.46 (0.43-0.51)	0.59 (0.53-0.65)	0.61 (0.57-0.64)	0.65 (0.60-0.68)	0.62 (0.58-0.65)	0.22 (0.15-0.29)	0.00%	2.19
Phi-4-Mini-Instruct	0.43 (0.41-0.45)	0.87 (0.82-0.90)	0.56 (0.53-0.59)	0.40 (0.35-0.44)	0.63 (0.60-0.66)	0.27 (0.21-0.33)	0.00%	1.12
Qwen2.5-7B-Instruct	0.57(0.52-0.62)	0.66 (0.61-0.71)	0.69 (0.66-0.72)	0.74 (0.71-0.78)	0.70 (0.67-0.73)	0.39 (0.33- 0.45)	6.70%	2.13
Ministral-8B-Instruct-2410	0.44 (0.42-0.45)	0.94 (0.91-0.97)	0.56 (0.52-0.58)	0.36 (0.31-0.40)	0.65 (0.62-0.67)	0.33 (0.27-0.37)	0.00%	1.05
Llama-3.1-8B-Instruct	0.46 (0.44-0.49)	0.75 (0.70-0.81)	0.61 (0.58-0.64)	0.54 (0.49-0.58)	0.65 (0.61-0.68)	0.28 (0.22-0.34)	4.00%	3.13

Bold values highlight the best-performing model(s) for the selected key metric (Balanced Accuracy).

### Performance across model sizes

Understanding how model scale influences performance is important for selecting the most appropriate LLM in terms of both accuracy and efficiency. In this section, we analyze models of three different scales: small, mid-sized, and large.

Larger models ($\sim$70B parameters), such as Qwen-72B and Llama-3.1-Nemotron-70B, achieved the strongest average performance, with balanced accuracy around 0.80–0.81 and macro-F1 between 0.75–0.80. Mid-size models (24–32B) often matched or exceeded these results. Mistral-Small, for instance, reached macro-F1=0.80, precision=0.69, recall=0.82, and MCC=0.61, comparable to the Llama-70B variants. By contrast, Phi-3.5-MoE (24B) gave importance to recall (0.91) at the expense of precision (0.51), leading to a lower MCC of 0.44.

Smaller models, such as Llama-3.1-8B, Llama-3-8B, and Qwen-7B, showed more varied performance. Most small Llama variants clustered around macro-F1 scores of 0.71–0.74 and MCCs of 0.43–0.48, comparable to some 70B models but still behind mid-size models like Mistral-Small. Qwen-7B performed weaker, with macro-F1=0.65 and MCC=0.31, falling short of Qwen-72B. Ministral-8B was more modest, reaching macro-F1=0.55 and MCC=0.34.

We observe meaningful differences across scales, but improvements are not strictly linear. It is also worth noting that the number of mid-sized models in the evaluation is relatively small, which limits the strength of any broad generalizations about this category.

### Domain-specific, reasoning vs. generalist models

**Figure 7:**
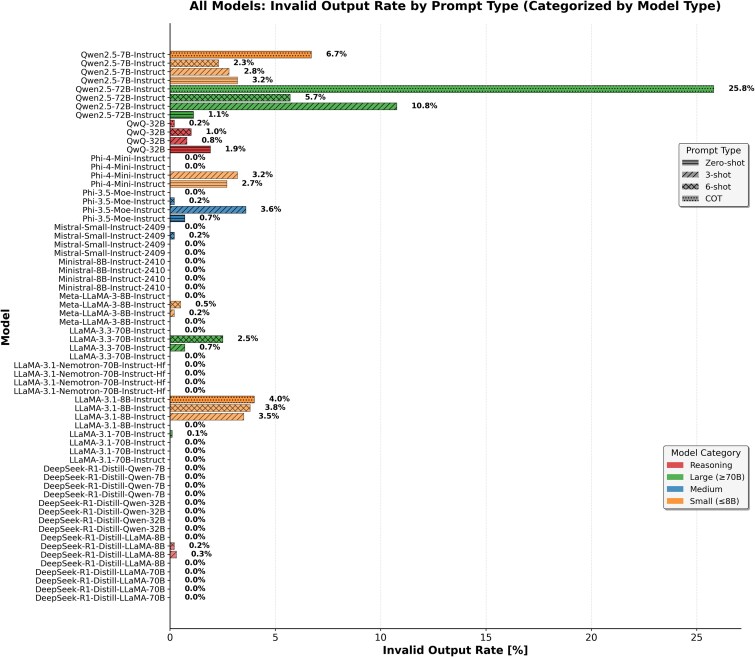
Invalid output rates across models and prompting strategies.

Domain-specific models, which were restricted to zero-shot due to context window limitations, showed polarized behaviors. BioMistral-7B and Meditron-70B gave importance to recall (e.g. BioMistral recall: 0.97), but suffered from extremely low specificity (as low as 0.10), limiting their overall balanced accuracy and MCC. In contrast, PMC-LLaMA reversed this trend, achieving a high specificity of 0.95 but near-zero recall at 0.05, resulting in a balanced accuracy of 0.50, MCC of 0.00, and a high invalid output rate of 17.10% (Fig. 7).

Interestingly, reasoning models demonstrated noticeable gains when evaluated under few-shot conditions. For instance, QwQ-32B improved from 0.68 to 0.78 in macro-F1, and from 0.39 to 0.59 in MCC under 6-shot prompting.

Overall, our trends show generalist models performing the best, particularly in zero-shot settings, but it is important to acknowledge that this comparison may not be entirely fair; many domain-specific models are built on older architectures and constrained by limited context windows. Moreover, even as other models struggled with few-shot prompting, reasoning models consistently benefited from it.

### Latency–performance trade-offs across prompting strategies

**Figure 8:**
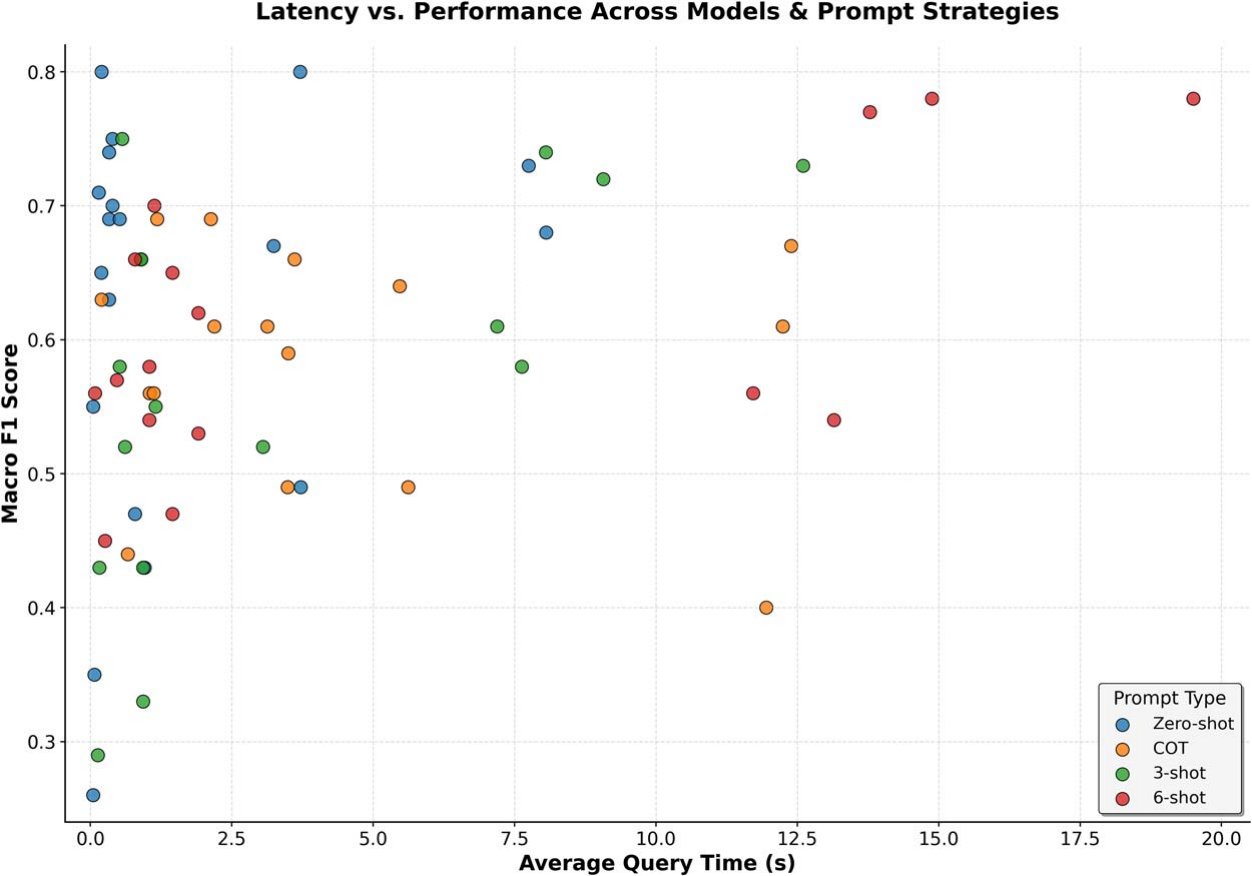
Latency versus performance (Macro-F1) across models and prompting strategies.

Here we address the question “What latency–performance trade-offs emerge across models and prompt types?”, summarized visually in Fig. 8 (latency vs. Macro-F1 scatter plot). This plot reveals that zero-shot delivers strong performance with the lowest latency. For example, Mistral-Small achieves Macro-F1 scores around 0.80 in under 0.3 seconds per query, making it highly efficient and effective at the same time. In contrast, few-shot prompting (3- and 6-shot) in most cases increases latency by 3–5$\times$, yet rarely improves accuracy and often degrades. CoT prompts further increase latency costs, while simultaneously lowering accuracy in most cases. The plot illustrates that many CoT and few-shot points shift rightward (slower) and downward (less accurate) compared to their zero-shot baselines.

### Performance robustness in strict vs. lenient evaluation

To evaluate the effect of stricter output parsing criteria on our model comparisons, we reran the zero-shot evaluation under a strict grading policy, where every invalid or malformed answer is treated as an incorrect prediction. As shown in Table 7, despite non-trivial invalid-output rates for some models (e.g. PMC-LLaMA at 17.1%, QwQ-32B at 1.91%), balanced accuracy in the zero-shot setting decreased by at most 1 pp, and the overall model ranking remained unchanged.

**Table 7:** Comparison of strict vs. lenient evaluation under zero-shot and 3-shot prompting.

**Prompt**	**Model**	**Balanced Acc. (Lenient)**	**Balanced Acc. (Strict)**	** $\Delta$ BA (pp)**	**Invalid Outputs (%)**
zero-shot	PMC-LLaMA	0.64	0.64	0.0	17.10%
	Meditron-70B	0.60	0.59	−1.0	2.52%
	QwQ-32B	0.71	0.70	−1.0	1.91%
	Qwen2.5-72B-Instruct	0.81	0.80	−1.0	1.11%
3-shot	Qwen2.5-72B-Instruct	0.75	0.74	−1.0	10.76%
	Phi-4-Mini-Instruct	0.52	0.51	−1.0	3.22%
	Phi-3.5-MoE-Instruct	0.64	0.64	0.0	3.62%
	Llama-3.1-8B-Instruct	0.57	0.57	0.0	3.52%

We additionally report 3-shot results as a stress test, motivated by their higher invalid-output rates. Even in this setting, models with more than 10% invalid outputs (e.g. Qwen-72B) experienced at most a 1 pp drop in balanced accuracy under strict evaluation. Overall, the performance differences observed under lenient evaluation are largely preserved under strict evaluation in the zero-shot setting, indicating that our core findings are robust to the choice of output parsing policy.

## Discussion

This study began with a rigorous exploration of the capabilities of LLMs in the classification of the existence of pathogen-disease relationships, given the title and abstract of a biomedical article. Our goal was to evaluate the efficiency and accuracy of multiple open-source foundational LLMs, which are of varying sizes ranging from 7B to 70B parameters, and establish their baseline capabilities. Following this, we intended to evaluate how different prompting techniques impacted the performance and efficiency. We also evaluated non-LLM baselines, including simple heuristics, a supervised TF–IDF + logistic regression classifier, and a fine-tuned BioBERT model. While these baselines provide useful context, they fall well short of the best zero-shot LLM performance.

Our findings are broadly consistent with the notion that, under certain conditions, larger models can offer performance advantages. That said, we did not observe a straightforward relationship between size and improvement. In our limited set of models, the step from roughly 7 billion to 13–24 billion parameters appeared to yield the most notable gains in balanced accuracy and macro-F1. In contrast, the increase from 33 billion to 70 billion parameters resulted in only modest or sometimes ambiguous improvements. Given the small and heterogeneous set of models evaluated, we do not suggest that this pattern is generalizable; rather, the results point to a more nuanced landscape, where architectural choices and other factors may play a significant role alongside model scale.

That said, size alone doesn’t guarantee reliability. Some of the largest models, such as Qwen-72B, scored very well on metrics like balanced accuracy and macro-F1, but these figures can be misleading. The model produced a high rate of invalid outputs, meaning many of its predictions could not be translated into predefined categories. As a result, its “high” scores are based on a reduced set of valid responses, potentially inflating its apparent performance; however, in our strict evaluation (counting invalid outputs as incorrect), balanced accuracy dropped by at most 1 pp and model rankings were unchanged, suggesting our conclusions are not sensitive to this effect. We nonetheless recommend reporting output validity alongside accuracy. Our error logs reveal three recurring failure modes behind these invalid outputs. (i) Instruction drift: models sometimes ignore the constrained {“relationship”: 1, “unrelated”: 0} schema and emit a free-text explanation. (ii) Code hallucination, especially in Qwen-72B under CoT prompting, where the model prints a Python function instead of a JSON-like dictionary. (iii) Parser mismatch: our regex-based extractor assumes the final dictionary in the output is the true answer. However, if the model echoes part of the prompt, including a dictionary, and then gets truncated mid-generation, the parser mistakenly captures the wrong dictionary. A promising fix would be to introduce a two-stage pipeline: the primary model generates a response, and a lightweight verifier LLM then classifies the output into expected categories.

Our experiments revealed a counterintuitive trend: increasing the number of shots (examples) often degraded performance across generalist models of varying sizes. We observed declines in precision, balanced accuracy, and the validity of the output as more context was added. Other studies suggest that additional context can enhance performance [23, 51]. The notion that excessive context can degrade outcomes contradicts the intuitive assumption that additional examples should enhance task understanding.

One possible explanation is that the addition of context through examples introduces greater complexity, which may dilute the model’s ability to focus on the core task. In models with limited context windows, excessive prompting may push relevant parts of the query further away from the model’s attention. It should also be noted, however, that our experiments did not control for possible confounding factors such as the quality of the examples themselves. As a result, we cannot confirm whether the observed pattern would persist under more tightly controlled conditions. These findings point to potential limitations and bring about the need for further investigation into prompt length and example quality.

However, reasoning models like QwQ-32B and distilled Deepseek variants defied this general trend, showing improved performance with more context. This difference likely reflects their inherent support for self-verification and structured reasoning principles closely aligned with CoT strategies. Our attempts to manually design CoT prompts, however, led to degraded performance. We believe the limits we observed likely stem from the deliberately simplistic, example-free prompting strategy we chose. By keeping prompts brief and omitting exemplars, we underutilized established CoT techniques. Future work should ideally explore richer, exemplar-based CoT prompts. As a result, these setbacks may not indicate an inherent limitation of the technique or the model itself. A valuable direction for future work would be to analyze the reasoning patterns exhibited by high-performing reasoning models like DeepSeek-R1 distills and QwQ-32B, particularly in how they structure their thinking steps. Studying these model-generated chains of thought, one could better understand the reasoning strategies they use and adapt from these structures to improve the design of our prompts.

To assess whether domain-specific pretraining on biomedical or clinical corpora would provide an advantage for this classification task, we evaluated BioMistral-7B, PMC-LLaMA, and Meditron-70B; however, our evaluations were restricted solely to zero-shot mode because their short context windows made few-shot or CoT prompting impractical. BioMistral-7B was fast with strong recall, but PMC-LLaMA often produced malformed outputs, and Meditron-70B struggled to follow reasoning cues, so none outperformed the generalist baselines. Future work should revisit this analysis with newer biomedical LLMs that pair larger context windows with modern instruction-tuned architectures.

Turning to the dataset, models performed well on abstracts with a clear structure, particularly those that explicitly stated their findings, such as “We investigated X and found Y”. They also performed well when a significant relationship between a pathogen and disease was explicitly stated. However, performance dropped in cases where the relationship was weakly defined, negative, or buried in complex subclauses. The models struggled to identify relationships in abstracts where the cohort was linking the pathogen and disease, e.g. the cohort consists of patients with a disease; later in the results, the pathogen was mentioned with the (abbreviated) cohort. Relationships in abstracts mentioned to be “not significant”, or mentioned to be not causal were less likely to be identified as having a relationship, indicating that LLMs tend to bias positive relationships more than neutral or negative relationships. Another pitfall for LLMs was pathogen or disease names in the article containing the disease or pathogen of the prompt, e.g. *Japanese encephalitis virus* containing the disease encephalitis. Both general and bio-specific LLMs seem not to have distinguished between the pathogen and disease occurrence of encephalitis, leading to falsely identifying relationships. Abstracts with multiple pathogen–disease pairs or highly condensed biomedical language caused further confusion, often leading the models to hallucinate or incorrectly pair terms based on proximity rather than context.

The evaluated models were pretrained on large-scale web and scientific corpora that likely include PubMed abstracts, and complete control over memorization is therefore not possible. To mitigate leakage effects, we framed the task strictly as title and abstract-only binary classification, explicitly instructing models to rely solely on the provided text rather than external knowledge. The dataset spans diverse pathogens and diseases, reducing the likelihood that memorized associations systematically explain performance. Moreover, frequent errors on negated, non-significant, or structurally complex abstracts suggest that model behavior reflects limitations in textual interpretation rather than recall of known pathogen–disease facts.

Latency plays a critical role in model selection, especially when LLMs are deployed in time-sensitive downstream tasks. Our evaluation shows that smaller models such as Mistral-Small and Ministral-8B achieved remarkably low inference times (0.05–0.20s), while still performing competitively in zero-shot classification. Notably, some small models approached or even matched the performance of significantly larger counterparts, indicating that latency and model size do not always correlate with performance. Ultimately, model selection should come down to how much latency, accuracy, and output robustness matter for the specific downstream classification task.

As detailed in the Results section, a paired-bootstrap analysis showed that Mistral-Small and Llama-3.1-Nemotron-70B are statistically indistinguishable on $\Delta$-balanced accuracy, whereas Qwen2.5-72B lags behind. We therefore treat the former two as a single top-performance band and focus selection on latency and cost. While both Nemotron and Mistral deliver strong accuracy, they differ substantially in model scale: Nemotron is a full 70B-parameter model, while Mistral-Small is just 24B. Despite this, their inference times differ by only a few seconds. Which model is preferable may depend on the downstream task’s tolerance for model size and associated resource constraints.

## Limitations

Here, we acknowledge a few limitations in our setup. Firstly, our ground truth relies on human-curated labels to determine whether a given abstract establishes a relationship between a pathogen and a disease. While necessary, this may introduce a potential bias, especially given that we are working with existing abstracts not originally written for this specific task. This is reflected in the Inter-annotator agreement Cohen’s $\kappa$ being 0.50 (moderate consistency) between the independent evaluations of the dataset, and errors in labeling or inconsistencies in how disagreements were resolved during the original curation process could influence both model evaluation and the conclusions we draw, highlighting the subjective nature of abstract-level pathogen-disease relationship assessment. Another fundamental constraint is that all models operated on abstracts and titles alone. In real-world biomedical research, important contexts that could confirm or reject a relationship between a pathogen and a disease may be buried deeper in the full text, perhaps within methods, results, or discussion sections. By focusing solely on abstracts, we risk missing or misclassifying nuanced relationships, especially in ambiguous cases.

Lastly, all models were evaluated using fixed generation parameters, with greedy decoding (temperature = 0) to ensure deterministic outputs for structured parsing. While this improved consistency, it may have limited the models’ ability to explore alternative completions. Future work could examine how different decoding settings impact performance for this task.

Looking ahead, future work could explore fine-tuning or ensemble methods to improve classification consistency. In this study, we avoided fine-tuning due to the limited dataset size and the risk of overfitting. However, with access to a larger and more diverse dataset, fine-tuning could become a viable strategy to improve model performance for our specific classification task.

## Conclusion

In summary, our evaluation revealed early signs of promise in using LLMs to identify pathogen–disease relationships within PAIS literature, particularly under zero-shot settings with well-structured abstracts. While overall performance remains inconsistent, models like Mistral-Small and Llama-3.1-Nemotron-70B demonstrated a strong trade-off between accuracy, inference speed, and reliability, highlighting the potential of smaller, efficient architectures in specialized classification tasks.

## Experimental prompts

**Figure 9:**
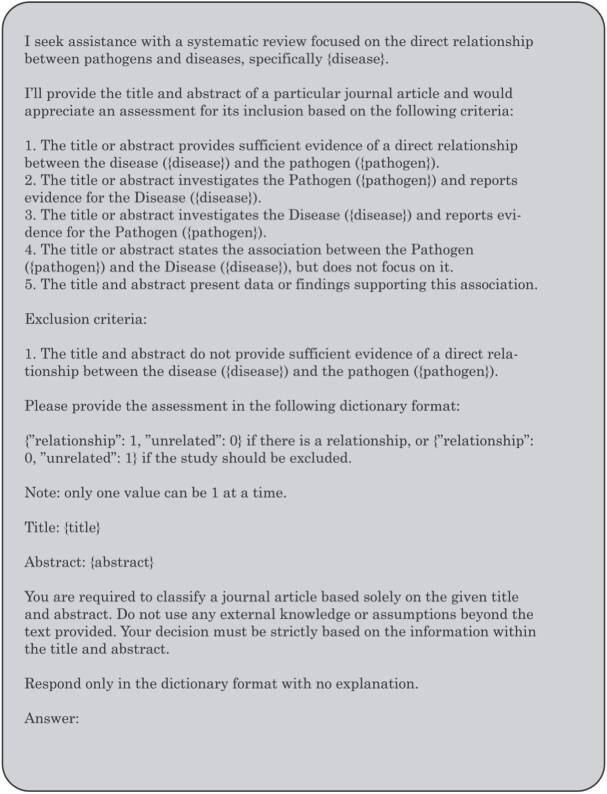
Full zero-shot prompt template used for pathogen–disease relationship classification.

**Figure 10:**
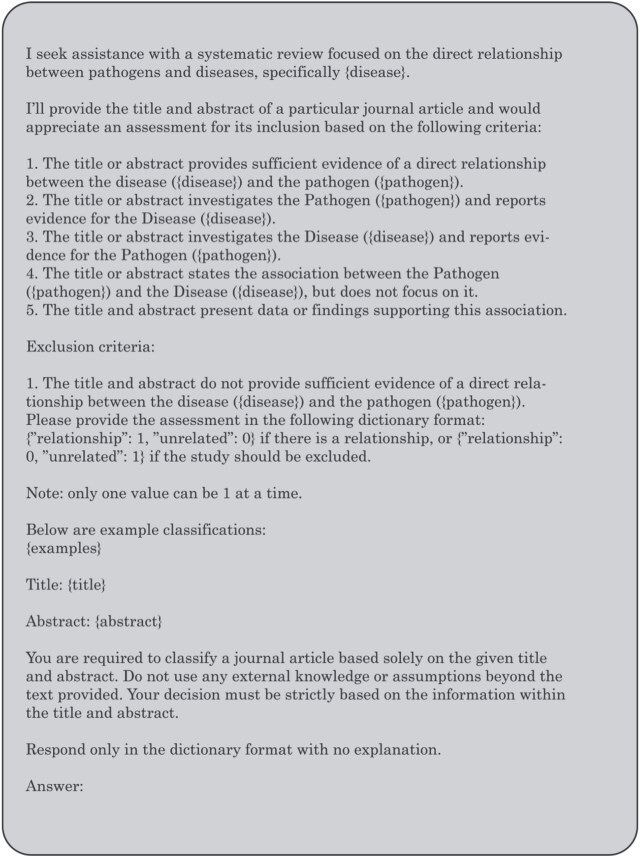
Full few-shot prompt template used for pathogen–disease relationship classification.

**Figure 11:**
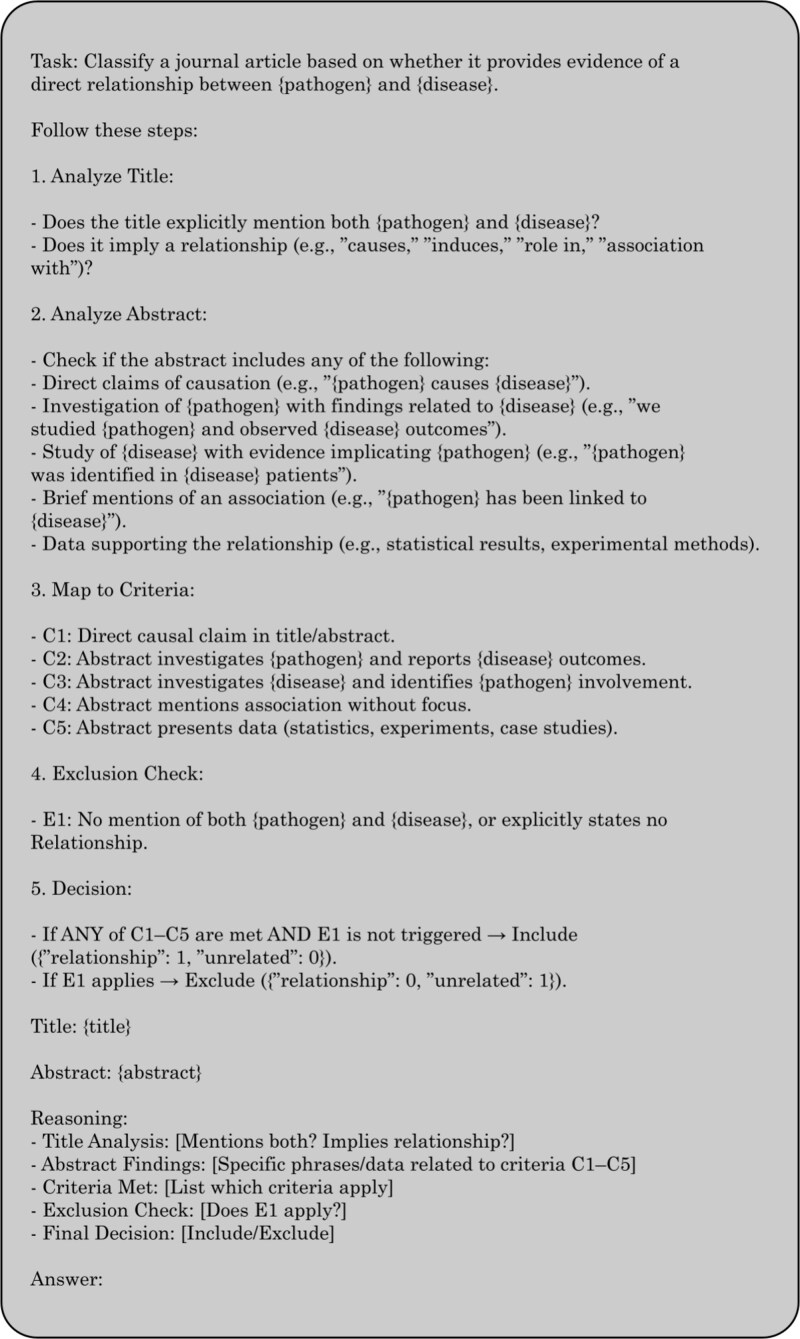
Full Chain-of-Thought prompt template used for pathogen–disease relationship classification.

The full text of the prompts used in our experiments is shown in Figs 9, 10, and 11.

Key Points

- This study benchmarks 19 open-weight large language models (LLMs) for identifying pathogen–disease relationships in post-acute infection syndromes (PAIS).
- A curated dataset of 1000 manually labeled PubMed abstracts was developed to evaluate pathogen–disease relationship classification.
- Zero-shot prompting provided the best balance of accuracy, reliability, and output validity across models.
- The large model **Llama-3.1-Nemotron-70B-Instruct** and the mid-sized **Mistral-Small-Instruct-2409 (22B)** achieved the highest balanced accuracy ($\approx$0.81) and macro-F1 scores ($\approx$0.80).
- Few-shot prompting generally degraded performance in generalist models but improved reasoning models like **DeepSeek-R1-Distill-Llama-70B** and **QwQ-32B**.

## Data Availability

The dataset and code supporting this study are publicly available via Zenodo. The dataset includes only PubMed metadata and labels due to copyright restrictions.
